# Asciminib in patients with newly diagnosed chronic myeloid leukemia: results from the Japanese subgroup of ASC4FIRST

**DOI:** 10.1007/s12185-025-04014-z

**Published:** 2025-05-26

**Authors:** Naoto Takahashi, Yoshikane Kikushige, Hirohisa Nakamae, Tatsunori Goto, Akihiro Tomita, Michiko Ichii, Satoshi Ito, Takanori Teshima, Keita Kirito, Takayuki Ikezoe, Kaoru Hatano, Hirokazu Tanaka, Nobuhiro Hiramoto, Ryohei Osako, Makoto Aoki, Kamel Malek, Yasunori Ueda

**Affiliations:** 1 Department of Hematology, Nephrology, and Rheumatology, Akita University School of Medicine, Akita, Akita Japan; 2 Center for Cellular and Molecular Medicine, Kyushu University Hospital, Fukuoka, Fukuoka Japan; 3 Department of Hematology, Osaka Metropolitan University Hospital, Osaka, Osaka Japan; 4 Department of Hematology, Japanese Red Cross Aichi Medical Center Nagoya Daiichi Hospital, Nagoya, Aichi Japan; 5 Department of Hematology, Fujita Health University School of Medicine, Toyoake, Aichi Japan; 6 Department of Hematology and Oncology, Osaka University Hospital, Suita, Osaka Japan; 7 Department of Hematology, Yamagata University Hospital, Yamagata, Yamagata Japan; 8 Department of Hematology, Hokkaido University Faculty of Medicine, Sapporo, Hokkaido Japan; 9 Department of Hematology and Oncology, University of Yamanashi, Chuo, Yamanashi Japan; 10 Department of Hematology, Fukushima Medical University, Fukushima, Fukushima Japan; 11 Division of Hematology, Department of Medicine, Jichi Medical University, Shimotsuke, Tochigi Japan; 12 Department of Hematology and Rheumatology, Kindai University Faculty of Medicine, Osaka-Sayama, Osaka Japan; 13 Department of Hematology, Kobe City Medical Center General Hospital, Kobe, Hyogo Japan; 14 Novartis Pharma K.K., Tokyo, Japan; 15 Novartis Pharma AG, Basel, Switzerland; 16 Department of Hematology/Oncology, Kurashiki Central Hospital, Kurashiki, Okayama Japan

**Keywords:** Asciminib, TKI, CML, First-line, Japanese

## Abstract

**Introduction:**

The phase III ASC4FIRST study (NCT04971226) demonstrated superior efficacy and favorable safety and tolerability for asciminib against investigator-selected tyrosine kinase inhibitors (IS-TKI) in newly diagnosed chronic myeloid leukemia (CML). Results of a subgroup analysis in Japanese patients are presented here.

**Methods:**

Adult patients were randomized 1:1 to asciminib or IS-TKI following stratification by European Treatment and Outcome Study long-term survival risk score and prerandomization-selected TKI (imatinib and second-generation [2G] TKI strata). At week 48, major molecular response (MMR) rate in all patients and imatinib stratum (primary endpoints) were assessed along with MR4.0, MR4.5, and safety (cutoff: November 28, 2023).

**Results:**

In Japanese patients (asciminib, n = 21; IS-TKI, n = 17 [imatinib/2G TKI, n = 8/9]), the MMR rate was higher with asciminib (81.0%) than IS-TKI (47.1%), and versus imatinib (asciminib: 100%; imatinib: 25.0% [imatinib stratum]). More patients on asciminib than IS-TKI achieved MR4.0 (57.1% vs. 11.8%) and MR4.5 (28.6% vs. 5.9%). Fewer grade ≥ 3 adverse events (AEs; 42.9%, 50.0%, and 55.6%) and AEs leading to treatment discontinuation (0%, 37.5%, and 11.1%) occurred with asciminib than imatinib or 2G TKI.

**Conclusion:**

Outcomes in Japanese patients were consistent with the ASC4FIRST overall population. Asciminib may be a therapy of choice for Japanese patients with CML.

**Supplementary Information:**

The online version contains supplementary material available at 10.1007/s12185-025-04014-z.

## Introduction

The first-generation tyrosine kinase inhibitor (TKI), imatinib, and subsequent second-generation (2G) TKIs, nilotinib, dasatinib and bosutinib have all demonstrated efficacy in patients with newly diagnosed chronic myeloid leukemia (CML) in chronic phase (CP) [1–4]. As such, these agents are recommended by international treatment guidelines (including the National Comprehensive Cancer Network^®^ [NCCN^®^], European LeukemiaNet [ELN], and the Japanese Society of Hematology) as the first-line treatment for newly diagnosed CML-CP [5–8]. Patients with CML who are treated with a TKI can expect a 5-year overall survival rate that is only slightly lower than that of the general population [9]. However, too many patients who receive currently available TKIs fail to meet treatment goals and discontinue or change therapies within the first year, with further switching in subsequent therapy lines; this occurs most often due to intolerance and the development of resistance (including ABL kinase mutations) [10–15]. Sequential treatment changes are associated with poorer outcomes, including development of resistance, lower response rates, higher risk of progression, and lower overall survival [10, 11].

In Japan, four TKIs (imatinib, nilotinib, dasatinib, and bosutinib) are currently available for first-line treatment of patients with CML [6]. The current Japanese practical guidelines recommend all four TKIs for patients with newly diagnosed CML-CP and the most appropriate treatment option should be based on the individual patient’s risk and background, including disease characteristics and comorbidities [6]. Although several studies have demonstrated favorable clinical outcomes with TKIs in Japanese patients with newly diagnosed CML-CP [16–19], there is still a need for treatments that are both highly efficacious and well tolerated to enable more patients to achieve their treatment goals, including treatment-free remission (TFR).

Asciminib is a first-in-class BCR::ABL1 inhibitor that works by specifically targeting the ABL myristoyl pocket (STAMP) and was intentionally designed to reduce off-target effects, while maintaining efficacy, compared to ATP-competitive TKIs [20]. The expected efficacy and safety benefits of asciminib have been demonstrated in the pivotal phase III ASCEMBL trial (NCT03106779), which showed the superior efficacy and favorable safety profile of asciminib compared with the 2G TKI bosutinib in patients with CML-CP previously treated with ≥ 2 TKIs [21]. These benefits were sustained for up to 96 weeks of follow-up [22]. This outcome was mirrored in the Japanese subgroup of ASCEMBL, where the efficacy and safety of asciminib were consistent with that of the overall study population at week 24 and maintained up to week 96 [23, 24]. Based on the results of a phase I dose-finding trial (NCT02081378) conducted in patients with T315I mutation [25] and ASCEMBL [21], asciminib was approved in the USA and Europe for the treatment of patients with CML-CP previously treated with ≥ 2 TKIs, and additionally in the USA for patients harboring the T315I mutation [26, 27]. In Japan, asciminib was approved in 2022 for the treatment of patients with CML-CP and resistance or intolerance to previous TKI therapy [28] and is recommended in the current practical Japanese guidelines as a third-line or later treatment option [6].

ASC4FIRST (NCT04971226) is the first phase III trial to evaluate the efficacy and safety of asciminib versus investigator-selected TKIs, which includes the current standard-of-care TKIs (imatinib and 2G TKIs) in patients with newly diagnosed Philadelphia chromosome-positive (Ph+) CML-CP [29]. The primary analysis of ASC4FIRST demonstrated the statistically and clinically significantly superior efficacy and favorable safety and tolerability of asciminib compared with the current standard-of-care TKIs [29]. The primary endpoint of major molecular response (MMR, *BCR::ABL1* transcript levels on the International Scale [*BCR::ABL1*^IS^] ≤ 0.1%) rate at week 48 was significantly higher with asciminib compared with investigator-selected TKIs (67.7% versus 49.0%) with a difference (95% confidence interval, [CI]) of 18.9% ([9.6, 28.2]; p < 0.001). Similar outcomes were observed for MMR rate when asciminib was compared with imatinib (69.3% versus 40.2%, respectively; difference [95% CI] was 29.6% [16.9. 42.2]; p < 0.001). The MMR rate at week 48 was also numerically higher for asciminib (66.0%) than 2G TKIs (57.8%). In addition, asciminib had more favorable safety and tolerability than imatinib and 2G TKIs, with fewer patients experiencing both grade ≥ 3 adverse events (AEs; 38.0% of patients on asciminib versus 44.4% and 54.9% on imatinib and 2G TKI, respectively) and AEs leading to treatment discontinuation (4.5% of patients on asciminib compared with 11.1% and 9.8% of patients on imatinib and 2G TKI, respectively) [29]. Following these positive outcomes, asciminib received accelerated approval in the USA for the treatment of patients with newly diagnosed CML [30], expanding on its indications in previously treated CML and patients with CML harboring the T315I mutation [27].

Japan participated in and made an important contribution to the ASC4FIRST study. We present the subgroup analysis of Japanese patients from the ASC4FIRST study primary analysis to determine the efficacy and safety of asciminib in this patient subpopulation.

## Methods

### Ethics

The protocol was approved by institutional review boards at each study site and the study was conducted in accordance with the principles of the Declaration of Helsinki and the International Council for Harmonization Guidelines for Good Clinical Practice, with applicable local regulations. All patients provided informed consent.

### Study design and treatment

ASC4FIRST is an ongoing phase III, multicenter, open-label, randomized trial. Patients with newly diagnosed CML-CP were enrolled into the study from November 5, 2021, up to December 20, 2022. This analysis presents the results of patients randomized to treatment in the 14 Japanese institutions participating in the trial. All analyses are based on a data cutoff date of November 28, 2023, after patients had completed the week 48 visit or had discontinued treatment early.

The full study design, endpoints, assessments, and analyses have been reported previously [29]. In brief, patients were randomized 1:1 to receive either asciminib (80 mg once daily [QD]) or investigator-selected TKI (IS-TKI; given at the approved doses for first line [imatinib 400 mg QD, bosutinib 400 mg QD, dasatinib 100 mg QD, or nilotinib 300 mg twice daily [BID]). Before participants were randomized for treatment, investigators conferred with their patients and selected the preferred TKI that was best tailored to the individual patient (imatinib or one of the 2G TKIs evaluated in the study), should the patient be randomized to the comparator arm. Randomization was stratified by this prerandomization-selected TKI (into two different strata, imatinib, or 2G TKI) along with the patient’s European Treatment and Outcome Study (EUTOS) long-term survival score (ELTS) risk category (low, intermediate, and high) (Supplementary Fig. 1). Asciminib was given orally under fasting conditions.

During the study, a crossover of study treatment or change in study treatment in the IS-TKI arm was not allowed. Patients continued treatment until the end of the study (5 years from the last patient first treatment) or until discontinuation due to treatment failure, disease progression, intolerance, or patient/physician decision. Patients who discontinued treatment were followed up for survival and disease progression until the end of the study.

### Patients

The main eligibility criteria have been reported previously [29]. In summary, patients were aged ≥ 18 years with newly diagnosed Ph+ CML-CP according to ELN 2020 criteria [7] that had been diagnosed within 3 months before their enrollment in the study. Patients were excluded if they had received previous treatment for CML; treatment with hydroxyurea and/or anagrelide was allowed, as was imatinib, nilotinib, dasatinib, or bosutinib for up to 2 weeks before randomization, but not thereafter. Treatment with other anticancer agents prior to randomization was not permitted.

### Study objectives and endpoints

ASC4FIRST had two primary objectives. Firstly, to show the efficacy of asciminib against all IS- TKIs, and secondly to show the efficacy of asciminib against imatinib (imatinib stratum). The corresponding primary endpoints were MMR rates at week 48 for both. Patients who discontinued treatment for any reason or met any of the treatment failure criteria based on the ELN2020 criteria [7] before week 48 were considered not to have had a response.

Secondary efficacy endpoints included assessing MMR at week 48 for asciminib versus 2G TKIs (2G TKI stratum), molecular response (*BCR::ABL1*^*IS*^ transcript levels ≤ 1%, ≤ 0.01% [MR^4.0^], and ≤ 0.0032% [MR^4.5^] at week 48, and transcript level ≤ 10% at week 12), cumulative incidence rates for MMR, MR^4.0^, and MR^4.5^, and median time to MMR. *BCR::ABL1* mutational status was also assessed.

### Statistical analysis

Statistical testing was not performed for the Japanese subgroup outcomes and only descriptive statistics are provided. The efficacy analyses are reported as point estimates with 95% CI estimated using the Clopper–Pearson method, with the common treatment differences and their 95% CI estimated using the Mantel–Haenszel method after stratifying for prerandomization-selected TKI and baseline ELTS.

The cumulative response analysis was performed for molecular response using cumulative incidence functions for competing risk. The time to molecular response was defined as the time from the date of randomization to the date of the first documented occurrence of molecular response. Discontinuation of trial treatment for any reason (e.g., treatment failure or death) without attainment of the molecular response was considered a competing risk. The time to molecular response was censored at the last molecular assessment date during treatment, before or at the analysis cutoff, for patients who had not had a response or the competing risk event. The estimated cumulative incidence and 95% CI at the prespecified timepoints are presented.

Efficacy analyses for the Japanese subgroup were conducted in the (1) full analysis set, which comprised all Japanese patients randomized to treatment (all asciminib versus all IS-TKI); (2) the imatinib stratum full analysis set, which included those patients whose prerandomization-selected TKI was imatinib; and (3) 2G TKI stratum full analysis set, which included patients whose prerandomization-selected TKI was a 2G TKI (nilotinib, dasatinib, or bosutinib). Safety analyses were conducted in the safety analysis set, which included all Japanese patients who received at least one dose of study treatment (analyzed according to the actual treatment received). AEs were coded using Medical Dictionary for Regulatory Activities (MedDRA; version 26.1) and assessed according to Common Terminology Criteria for Adverse Events (CTCAE; version 5.0).

## Results

### Patients

Thirty-eight patients from 14 sites in Japan were enrolled and included in the Japanese subgroup analysis. The baseline characteristics of these patients were well balanced across arms. Median (range) age was 57.0 (18.0–74.0) years and 54.0 (41.0–76.0) years in Japanese patients in the asciminib and IS-TKI arms, respectively. The majority of patients were male (76.2% and 76.5% for asciminib and IS-TKI, respectively). In addition, 81.0% and 70.6% of patients in the asciminib and IS-TKI arms, respectively, had a low ELTS score (Table 1; full baseline characteristics in Supplementary Table 1), which is a higher proportion than that observed in the overall study population.

**Table 1:** Patient disposition and demographics in the Japan subgroup

	Asciminib			IS-TKI		
	Imatinib (n = 10)	2G TKI (n = 11)	All asciminib (n = 21)	Imatinib (n = 8)	2G TKI (n = 9)	All comparators (n = 17)
Patient disposition						
Patients randomized, n (%)	10 (100)	11 (100)	21 (100)	8 (100)	9 (100)	17 (100)
Patients treated^a^, n (%)	10 (100)	11 (100)	21 (100)	8 (100)	9 (100)	17 (100)
Treatment ongoing^b^, n (%)	10 (100)	9 (81.8)	19 (90.5)	3 (37.5)	8 (88.9)	11 (64.7)
Discontinued treatment, n (%)	0	2 (18.2)	2 (9.5)	5 (62.5)	1 (11.1)	6 (35.3)
Reason for discontinuation^c^, n (%)						
AE	0	1 (9.1)^d^	1 (4.8)^d^	3 (37.5)	1 (11.1)	4 (23.5)
Unsatisfactory therapeutic effect	0	1 (9.1)	1 (4.8)	1 (12.5)	0	1 (5.9)
Other	0	1 (9.1)	1 (4.8)	1 (12.5)	0	1 (5.9)
Progressive disease	0	0	0	1 (12.5)	0	1 (5.9)
Patient demographics						
Median (range) age, years	60.5 (41.0–74.0)	54.0 (18.0–61.0)	57.0 (18.0–74.0)	59.0 (41.0–76.0)	54.0 (42.0–72.0)	54.0 (41.0–76.0)
Male/female, n (%)	9 (90.0)/1 (10.0)	7 (63.6)/4 (36.4)	16 (76.2)/5 (23.8)	5 (62.5)/3 (37.5)	8 (88.9)/1 (11.1)	13 (76.5)/4 (23.5)
ELTS score (IRT), n (%)						
Low	8 (80.0)	9 (81.8)	17 (81.0)	6 (75.0)	6 (66.7)	12 (70.6)
Intermediate	2 (20.0)	0	2 (9.5)	2 (25.0)	3 (33.3)	5 (29.4)
High	0	2 (18.2)	2 (9.5)	0	0	0

*2G* second generation, *AE* adverse event, *ELTS* European Treatment and Outcome Study (EUTOS) long-term survival score, *IRT* interactive response technology, *IS-TKI* investigator-selected tyrosine kinase inhibitor, *n* represents patient numbers

^a^One patient stratified to imatinib as prerandomization selection of TKI received nilotinib; therefore, this patient is counted in the imatinib stratum for efficacy and in the 2G TKI stratum for safety analysis

^b^At data cutoff (November 28, 2023)

^c^Patients may have multiple reasons for discontinuing treatment

^d^This patient discontinued due to an AE more than 30 days after the last dose of asciminib. AEs that did not occur during treatment or within 30 days of the patient’s last study medication were not considered as ‘on treatment’

Of the 38 patients who participated, 21 received asciminib (imatinib stratum, n = 10; 2G TKI stratum, n = 11) and 17 received IS-TKI (imatinib stratum, n = 8; 2G TKI stratum, n = 9 [nilotinib, n = 2; dasatinib, n = 3; bosutinib, n = 4]) (Table 1). As of the data cutoff, treatment was ongoing in 19 (90.5%) and 11 (64.7%) patients in the asciminib and IS-TKI arms, respectively. A total of 2 (9.5%) and 6 (35.3%) patients discontinued asciminib and IS-TKI, respectively. IS-TKI discontinuations were primarily due to AEs (n = 4; 23.5%), whereas in the asciminib arm only 1 patient cited an AE as the reason for discontinuing treatment. However, it should be indicated that this patient discontinued more than 30 days after the last actual administration of asciminib, and therefore, per protocol, the discontinuation of this patient was not considered as occurring during the ‘on treatment’ period (Table 1).

### Efficacy

**Fig. 1:**
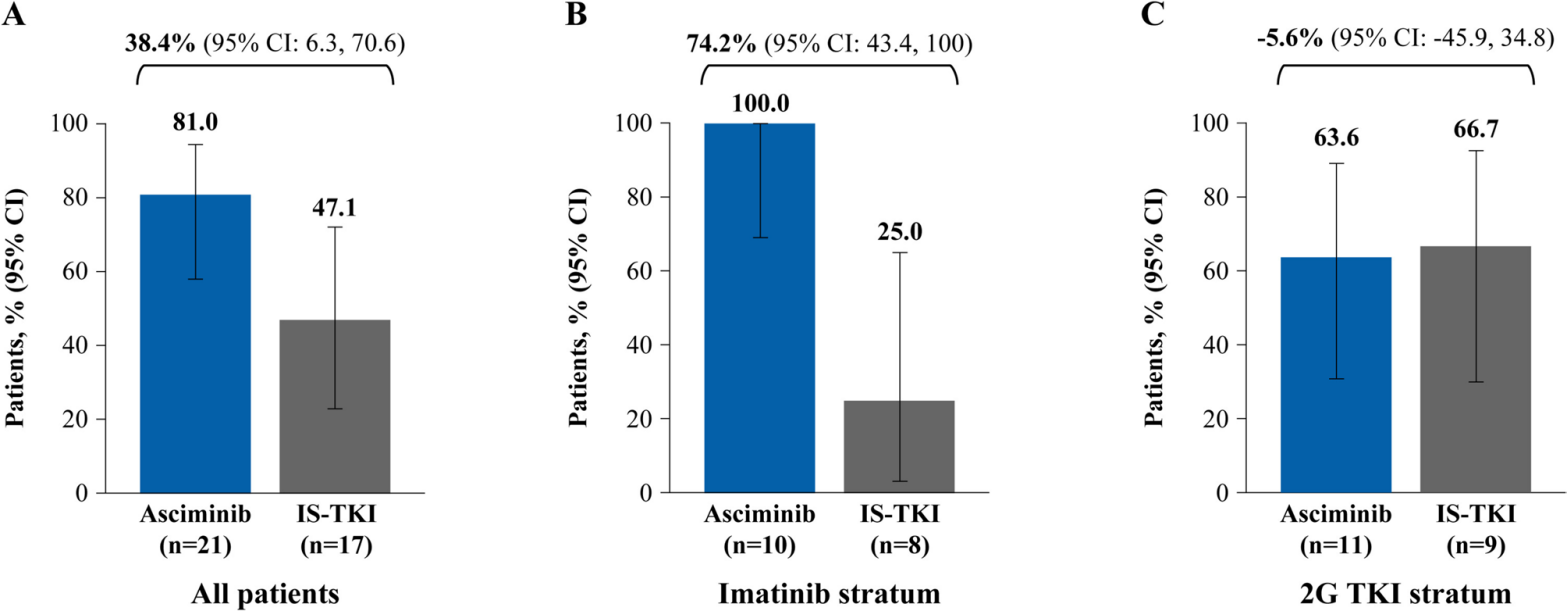
MMR at week 48 in the asciminib and IS-TKI arms in **A** all patients, **B** imatinib stratum, and **C** 2G TKI stratum. *2G* second generation, *CI* confidence interval, *IS-TKI* investigator-selected tyrosine kinase inhibitor, *MMR* major molecular response, *TKI* tyrosine kinase inhibitor, *n* represents patient numbers in each arm. Common risk difference and 95% CI between the arms shown for full analysis group, imatinib and 2G TKI strata

MMR rate at week 48 was achieved by more patients on asciminib than IS-TKI (81.0% versus 47.1%, respectively), with a common risk difference (95% CI) of 38.4% (6.3, 70.6) (Fig. 1A). Similarly, in the imatinib stratum, all patients receiving asciminib achieved MMR at week 48 compared with a quarter of patients receiving imatinib (100.0% versus 25.0%, respectively; common risk difference [95% CI] 74.2% [43.4, 100.0]; Fig. 1B). In the 2G TKI stratum, a comparable proportion of patients in the two arms achieved MMR at week 48 (63.6% versus 66.7% for asciminib and 2G TKI, respectively; common risk difference [95% CI] − 5.6% [− 45.9, 34.8]; Fig. 1C).

**Fig. 2:**
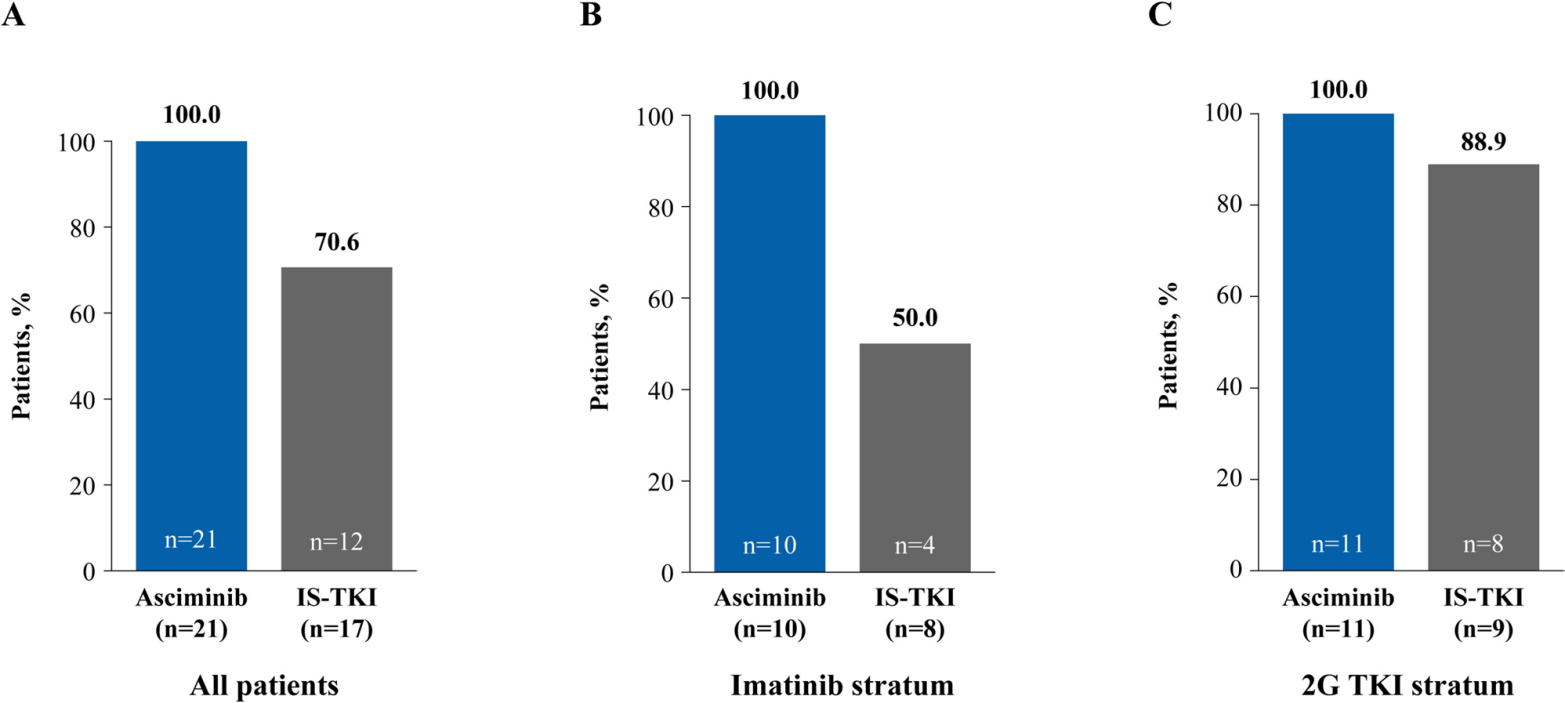
EMR at week 12 in the asciminib and IS-TKI arms in **A** all patients, **B** imatinib stratum, and **C** 2G TKI stratum. *2G* second generation, *EMR* early molecular response, *IS-TKI* investigator-selected tyrosine kinase inhibitor, *TKI* tyrosine kinase inhibitor; n represents patient numbers in the full analysis set for each arm. EMR is *BCR::ABL1*^IS^ ≤ 10% at week 12

**Fig. 3:**
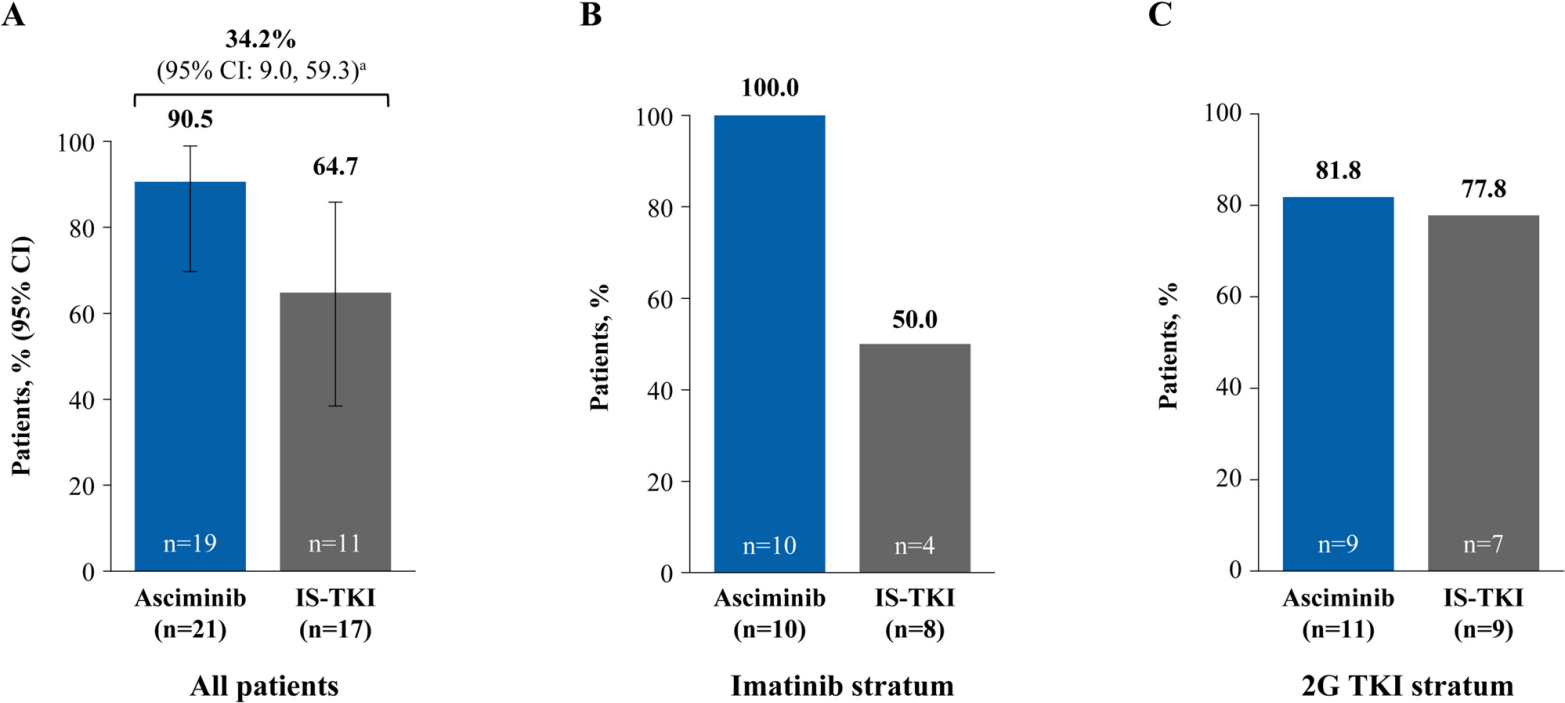
*BCR::ABL1*^IS^ ≤ 1% at week 48 in the asciminib and IS-TKI arms in **A** all patients, **B** imatinib stratum, and **C** 2G TKI stratum. *2G* second generation, *CI* confidence interval, *IS-TKI* investigator-selected tyrosine kinase inhibitor, *TKI* tyrosine kinase inhibitor, *n* represents patient numbers in the full analysis set for each arm. ^a^Common risk difference (95% CI) between the arms

**Fig. 4:**
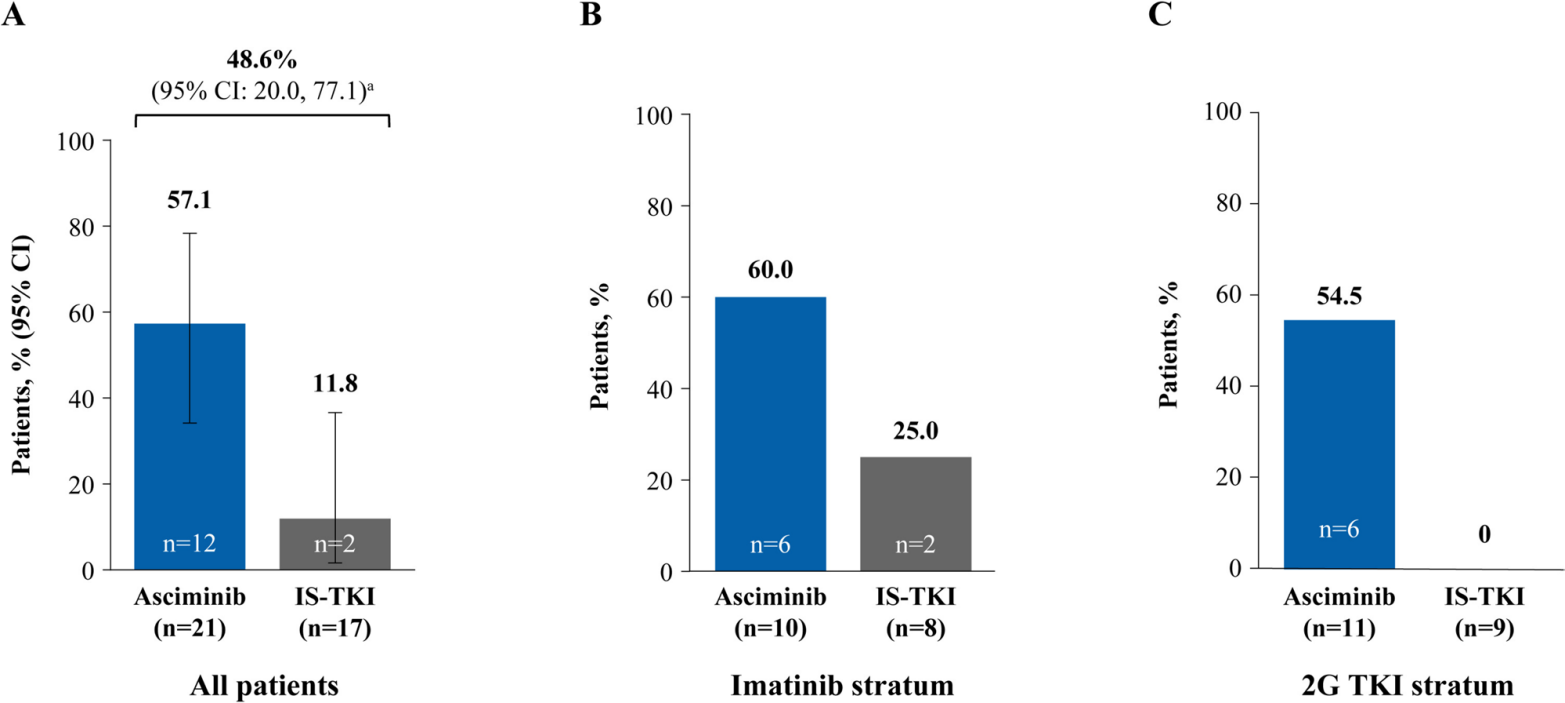
MR^4.0^ at week 48 in the asciminib and IS-TKI arms in **A** all patients, **B** imatinib stratum, and **C** 2G TKI stratum. *2G* second generation, *CI* confidence interval, *IS-TKI* investigator-selected tyrosine kinase inhibitor, *MR* molecular response, *TKI* tyrosine kinase inhibitor, *n* represents patient numbers in the full analysis set for each arm. ^a^Common risk difference (95% CI) between the arms

**Fig. 5:**
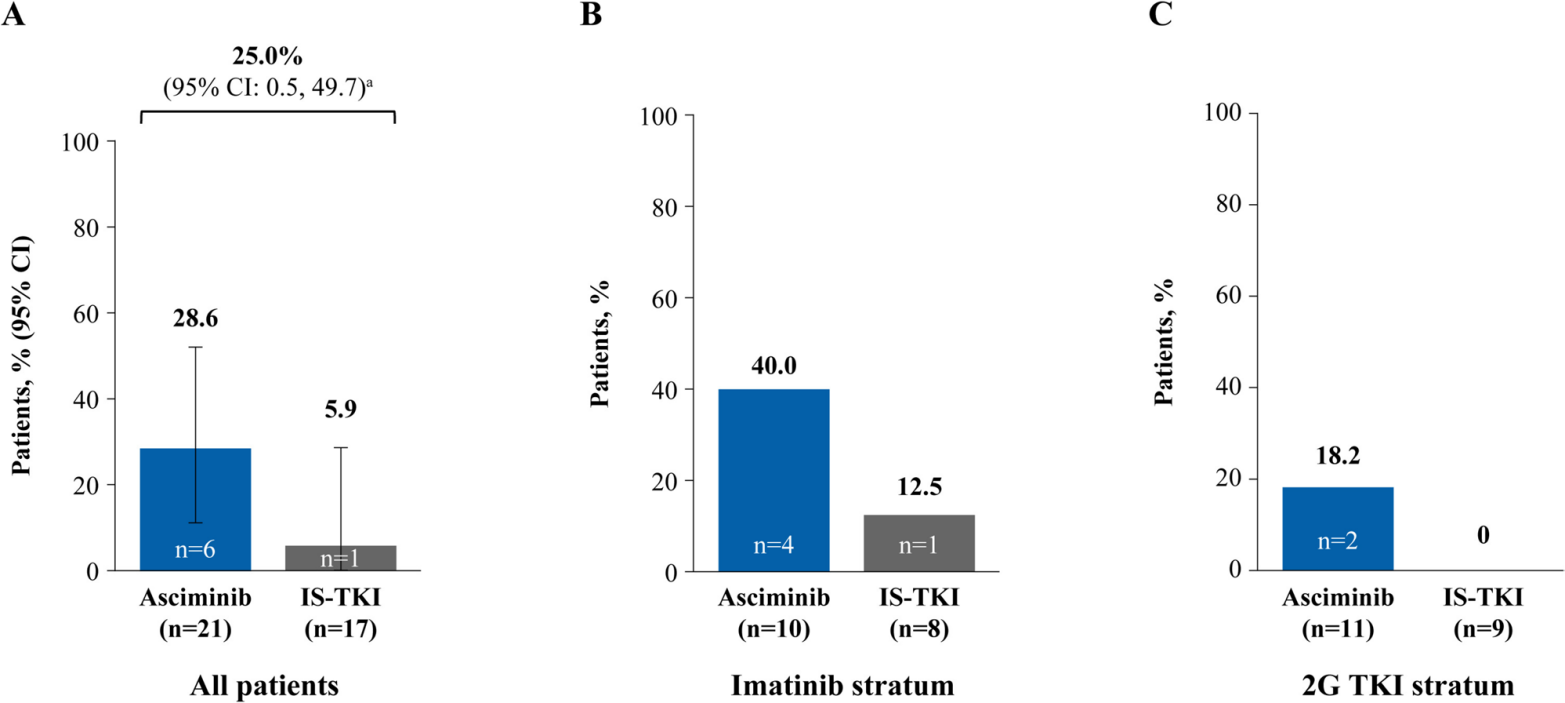
MR^4.5^ at week 48 in the asciminib and IS-TKI arms in **A** all patients, **B** imatinib stratum, and **C** 2G TKI stratum. *2G* second generation, *CI* confidence interval, *IS-TKI* investigator-selected tyrosine kinase inhibitor, *MR* molecular response, *TKI* tyrosine kinase inhibitor, *n* represents patient numbers in the full analysis set for each arm. ^a^Common risk difference (95% CI) between the arms

**Fig. 6:**
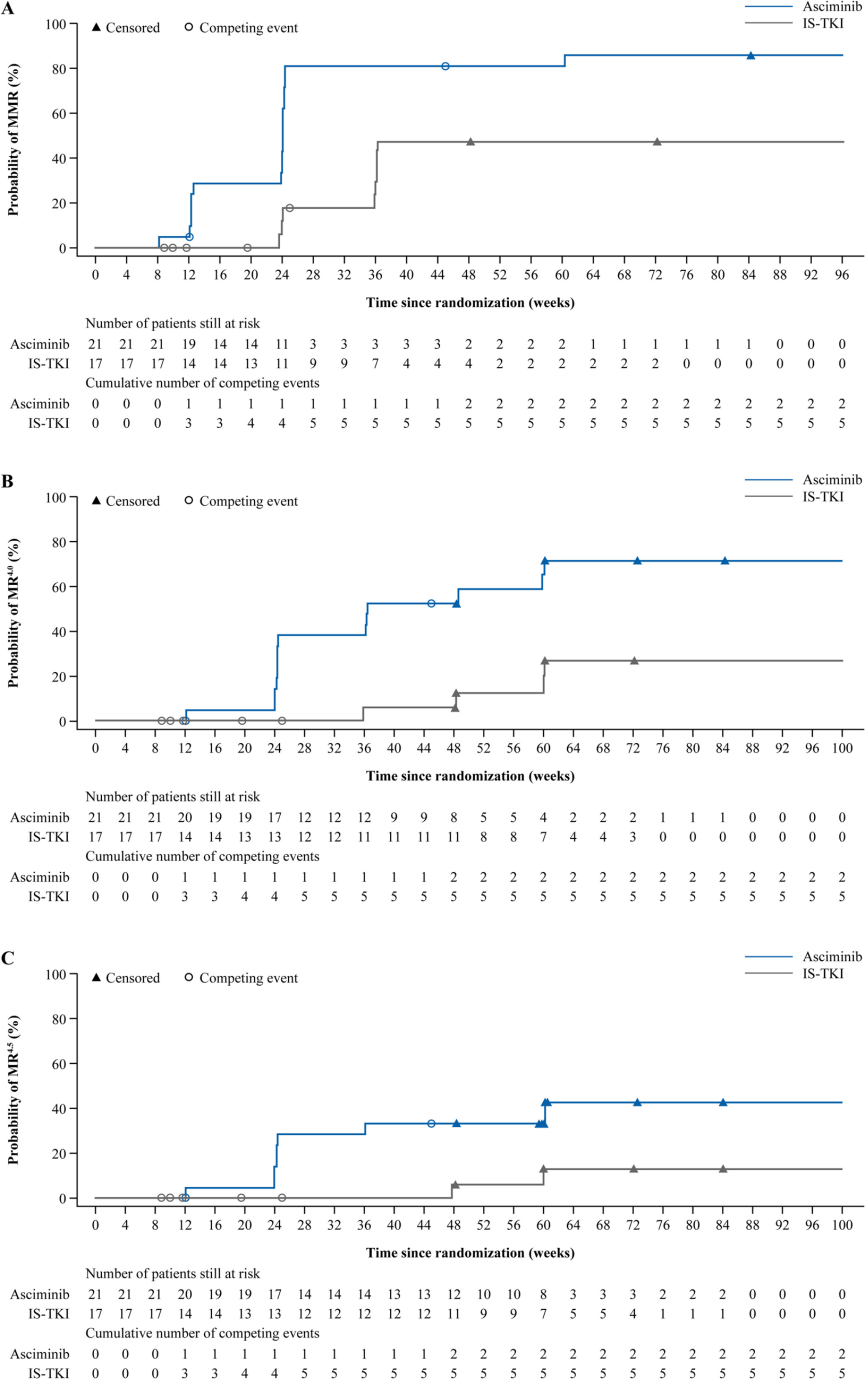
Cumulative incidence of **A** MMR, **B** MR^4.0^, and **C** MR^4.5^ in patients receiving asciminib or IS-TKI

All patients who received asciminib had an early molecular response (EMR; *BCR::ABL1*^IS^ ≤ 10% at week 12) compared with 70.6% of patients on IS-TKI, 50.0% on imatinib and 88.9% on 2G TKI (Fig. 2A, B and C). Similarly, more patients in the asciminib arm than the IS-TKI arm achieved a molecular response of *BCR::ABL1*^IS^ ≤ 1% at week 48 (90.5% and 64.7%, respectively; Fig. 3A), which was also reflected in patients who received imatinib and 2G TKI (Fig. 3B and C). Notably, deep molecular responses were also achieved by more patients on asciminib than IS-TKI (MR^4.0^ at week 48: 57.1% and 11.8%, respectively; MR^4.5^ at week 48: 28.6% and 5.9%, respectively; Figs. 4A and 5A) and numerically higher percentages of patients on asciminib achieved MR^4.0^ and MR^4.5^ at week 48 than patients who received imatinib (Figs. 4B and 5B, respectively) and 2G TKIs (Figs. 4C and 5C, respectively). For patients receiving asciminib, there was a higher probability of achieving MMR, MR^4.0^, and MR^4.5^ compared with patients receiving IS-TKI (Fig. 6). By week 48, the cumulative incidence rates for MMR in the asciminib and IS-TKI arms were 81.0% versus 47.1%, respectively; for MR^4.0^, they were 52.4% versus 5.9%, respectively, and for MR^4.5^, they were 33.3% versus 5.9%, respectively. MMR was reached earlier in patients receiving asciminib, with median (95% CI) times to MMR of 24.1 (12.6, 24.3) and 36.1 (24.1, not estimable [NE]) weeks in the asciminib and IS-TKI arms, respectively.

In this population of newly diagnosed patients with CML, over the course of treatment newly emerging *BCR::ABL1* mutations were identified in 1 patient only (asciminib arm), which were detected in or near the myristoyl pocket (A337T/L340Q) at week 41. This patient (male and 58 years of age) had *BCR::ABL1*^IS^ 66.0% at baseline, which reduced to 0.59% by week 12 indicating early molecular response, and to 0.24% by week 24 of asciminib treatment at which time the patient was still mutation free. At week 41, *BCR::ABL1*^IS^ (%) had risen to 2.2% and mutations A337T and L340Q were detected. At week 45, *BCR::ABL1*^IS^ was 4.8% and the patient discontinued study treatment due to unsatisfactory therapeutic effect, before meeting the ELN criteria for treatment failure.

### Safety

The median (range) duration of exposure to asciminib was 67.3 (5.9–94.1) weeks. Similarly, for all IS-TKI, the median (range) exposure was 68.9 (4.0–86.7) weeks, which included 39.4 (7.9–77.1) weeks of exposure to imatinib and 73.1 (4.0–86.7) weeks for 2G TKI. Notably, 90.5% of patients on asciminib and 70.6% of patients on IS-TKI had ≥ 48 weeks of treatment exposure. A relative dose intensity of between > 90% and 110% was reported in 95.2% of patients receiving asciminib and in 47.1% of patients receiving IS-TKI.

At least 1 AE (any grade) was reported in all patients who received asciminib, imatinib and 2G TKI, whereas fewer grade ≥ 3 AEs were reported with asciminib (42.9%) compared to imatinib and 2G TKI (50.0% and 55.6%, respectively; Supplementary Table 2). The most common grade ≥ 3 AEs were primarily hematological in nature, including thrombocytopenia (14.3%, 0%, and 11.1%), neutropenia (19.0%, 12.5%, and 11.1%, respectively) and lymphopenia (4.8%, 25.0%, and 0%, respectively) for asciminib, imatinib, and 2G TKI, respectively (Table 2). No arterial-occlusive events (AOEs) were reported in the Japanese subgroup. The percentage of patients with treatment-related AEs was lower with asciminib (76.2%) than imatinib and 2G TKI (both 100%; Supplementary Table 2). Similarly, there was a lower percentage of patients with AEs leading to dose adjustment/interruption with asciminib (33.3%) than imatinib (62.5%) and 2G TKI (66.7%); these were mainly hematological for asciminib (neutropenia, n = 4; thrombocytopenia, n = 3) and imatinib (neutropenia, n = 2; leukopenia, n = 2), and for 2G TKI were diarrhea and alanine aminotransferase increased (n = 2 each). Notably, no events of AEs leading to treatment discontinuation were reported with asciminib, whereas 3 patients (37.5%) on imatinib and 1 patient (11.1%) who received 2G TKI discontinued treatment due to AEs; AEs leading to treatment discontinuation were diarrhea, pyrexia, lymphopenia, and prostate cancer (imatinib; n = 1 for each AE), and thrombocytopenia (2G TKI; n = 1). As of the data cutoff, no deaths had occurred during the treatment period. One patient died in the imatinib arm (12.5%) due to CML during the survival follow-up period.

**Table 2:** Most common AEs and grade ≥ 3 AEs occurring in at least 3 patients in any treatment arm

AE in ≥ 3 patients^a^, n (%)	Asciminib		IS-TKI					
	All asciminib (n = 21)		Imatinib (n = 8)		2G TKI (n = 9)		All comparators (n = 17)	
	All grades	Grade ≥ 3	All grades	Grade ≥ 3	All grades	Grade ≥ 3	All grades	Grade ≥ 3
Thrombocytopenia^b^	9 (42.9)	3 (14.3)	5 (62.5)	0	3 (33.3)	1 (11.1)	8 (47.1)	1 (5.9)
Neutropenia^b^	9 (42.9)	4 (19.0)	5 (62.5)	1 (12.5)	1 (11.1)	1 (11.1)	6 (35.3)	2 (11.8)
Leukopenia^b^	7 (33.3)	0	4 (50.0)	1 (12.5)	1 (11.1)	1 (11.1)	5 (29.4)	2 (11.8)
Anemia	6 (28.6)	0	2 (25.0)	0	2 (22.2)	0	4 (23.5)	0
Lymphopenia^b^	5 (23.8)	1 (4.8)	4 (50.0)	2 (25.0)	2 (22.2)	0	6 (35.3)	2 (11.8)
Diarrhea	5 (23.8)	0	3 (37.5)	0	3 (33.3)	1 (11.1)	6 (35.3)	1 (5.9)
Lipase increased	5 (23.8)	0	1 (12.5)	0	1 (11.1)	0	2 (11.8)	0
Amylase increased	5 (23.8)	0	0	0	1 (11.1)	0	1 (5.9)	0
COVID-19	4 (19.0)	0	4 (50.0)	0	3 (33.3)	0	7 (41.2)	0
Nasopharyngitis	4 (19.0)	0	1 (12.5)	0	1 (11.1)	0	2 (11.8)	0
Myalgia	3 (14.3)	0	1 (12.5)	0	1 (11.1)	0	2 (11.8)	0
Constipation	3 (14.3)	0	0	0	0	0	0	0
Oropharyngeal pain	3 (14.3)	0	0	0	0	0	0	0
ALT increase	1 (4.8)	0	0	0	3 (33.3)	1 (11.1)	3 (17.6)	1 (5.9)
Back pain	1 (4.8)	0	2 (25.0)	0	1 (11.1)	0	3 (17.6)	0
Headache	1 (4.8)	0	0	0	3 (33.3)	0	3 (17.6)	0
AST increase	0	0	0	0	3 (33.3)	0	3 (17.6)	0

AEs shown are those that occurred during treatment or within 30 days after receiving the last dose of study medication. A patient with multiple severity grades for an AE is counted under the maximum grade

*2G* second generation, *AE* adverse event, *ALT* alanine aminotransferase, *AST* aspartate aminotransferase, *IS-TKI* investigator-selected tyrosine kinase inhibitor, *n* number of patients

^a^AEs that occurred in ≥ 3 patients in any treatment arm (presented in descending order [%] based on asciminib group) and occurring during treatment or within 30 days of the last study medication received

^b^Thrombocytopenia includes thrombocytopenia and decreased platelet count; neutropenia includes neutropenia and decreased neutrophil count; lymphopenia includes lymphopenia and decreased lymphocyte count; leukopenia includes leukopenia and white blood cell count decrease. Medical Dictionary for Regulatory Activities (MedDRA; version 26.1); Common Terminology Criteria for Adverse Events (CTCAE; version 5.0)

## Discussion

ASC4FIRST is the first randomized, controlled study to compare asciminib with all current standard-of-care TKIs in patients with newly diagnosed CML, which allows a direct comparison of the efficacy and safety profiles of asciminib with these agents, including 2G TKIs. In the primary analysis of ASC4FIRST conducted in the overall study population, asciminib was shown to have superior efficacy and a favorable safety and tolerability profile compared with all current standard-of-care TKIs [29]. The current report is a subgroup analysis of ASC4FIRST and the first report of the efficacy and safety of asciminib in Japanese patients with newly diagnosed CML.

The efficacy outcomes from this Japanese subgroup analysis were consistent with those observed in the overall population. As per the primary analysis [29], the MMR rate at week 48 in Japanese patients was higher with asciminib than with both IS-TKI (81.0% versus 47.1%, respectively) and imatinib (100.0% versus 25.0%, respectively). When considered alongside that of the overall study population (67.7%) [29], the MMR rate of asciminib was numerically higher in the Japanese subgroup (81%) (Table 3). However, this outcome may have been influenced by the higher proportion of patients with low ELTS in the Japanese subgroup than in the overall study population (81.0% and 60.7%, respectively, in the asciminib arms) [29].

**Table 3:** Molecular responses and overall safety profiles in Japanese patients and all patients randomized to asciminib in ASC4FIRST

	Japan subgroup		All patients	
	Asciminib (n = 21)	IS-TKI (n = 17)	Asciminib (n = 201)	IS-TKI (n = 204)
Molecular response, n (%)				
MMR at week 48	17 (81.0)	8 (47.1)	136 (67.7)	100 (49.0)
Common risk difference, % (95% CI); p value	38.4 (6.3, 70.6)		18.9 (9.6, 28.2); p < 0.001	
EMR at week 12	21 (100.0)	12 (70.6)	180 (89.6)	143 (70.1)
*BCR::ABL1*^IS^ ≤ 1% at week 48	19 (90.5)	11 (64.7)	175 (87.1)	148 (72.5)
MR^4.0^ at week 48	12 (57.1)	2 (11.8)	78 (38.8)	42 (20.6)
MR^4.5^ at week 48	6 (28.6)	1 (5.9)	34 (16.9)	18 (8.8)

	Japan subgroup			All patients		
	Asciminib (n = 21)	IS-TKI (n = 17)		Asciminib (n = 200)	IS-TKI (n = 201)	
		Imatinib (n = 8)	2G TKI (n = 9)		Imatinib (n = 99)	2G TKI (n = 102)
AE category, n (%)						
All grade AEs	21 (100)	8 (100)	9 (100)	187 (93.5)	93 (93.9)	102 (100)
Grade ≥ 3 AEs	9 (42.9)	4 (50.0)	5 (55.6)	76 (38.0)	44 (44.4)	56 (54.9)
Treatment-related AEs	16 (76.2)	8 (100)	9 (100)	150 (75.0)	83 (83.8)	97 (95.1)
SAEs	2 (9.5)	1 (12.5)	1 (11.1)	22 (11.0)	12 (12.1)	20 (19.6)
AEs leading to discontinuation	0	3 (37.5)	1 (11.1)	9 (4.5)	11 (11.1)	10 (9.8)
AEs leading to dose adjustment/interruption	7 (33.3)	5 (62.5)	6 (66.7)	60 (30.0)	39 (39.4)	54 (52.9)
AEs requiring additional therapy	17 (81.0)	8 (100)	9 (100)	151 (75.5)	78 (78.8)	91 (89.2)

EMR is *BCR::ABL1*^IS^ ≤ 10% at week 12; MR^4.0^ is *BCR::ABL1*^IS^ ≤ 0.01%; MR^4.5^ is *BCR::ABL1*^IS^ ≤ 0.0032%. Patients with multiple severity grades for an AE are only counted under the maximum grade. Medical Dictionary for Regulatory Activities (MedDRA; version 26.1); Common Terminology Criteria for Adverse Events (CTCAE; version 5.0)

*AE* adverse event, *CI* confidence interval, *EMR* early molecular response, *IS-TKI* investigator selected tyrosine kinase inhibitor, *MMR* major molecular response, *SAE* serious AE, *n* represents number of patients

Complementing these positive MMR findings, Japanese patients also had a higher probability of attaining early and deep molecular responses with asciminib than with IS-TKIs. More Japanese patients achieved EMR, MR^4.0^ and MR^4.5^ with asciminib than with IS-TKIs (100% versus 70.6%, 57.1% versus 11.8%, and 28.6% versus 5.9%). This is consistent with the findings in the overall study population, although the percentages of Japanese patients achieving these responses were numerically higher [29]. When considering the efficacy of asciminib versus 2G TKI, the overall outcomes in Japanese patients are consistent with those in the overall study population [29], with numerically higher percentages of Japanese patients who achieved early (100% vs 88.9%) and deep (MR^4.0^, 54.5% versus 0%; MR^4.5^, 18.2% versus 0%) molecular responses with asciminib compared with 2G TKI, respectively. It is acknowledged that the deep molecular responses observed for 2G TKI were notably low compared with those of the overall population [29] and with previous TKI studies in Japanese patients [31, 32]. This may have been influenced by low patient numbers in the Japanese population, given that the responses with 2G TKI in the overall ASC4FIRST population were higher than in Japanese patients, but outcomes still favored asciminib: MR^4.0^ (35.0% versus 26.5%, respectively) and MR^4.5^ (16.0% versus 12.7%, respectively) in the asciminib arm versus 2G TKI arm, respectively [29]. Achieving EMR and MMR have been linked with long-term survival advantages for patients with CML [33]. For patients with newly diagnosed CML, not achieving EMR has been associated with lower rates of molecular response, higher risk of progression and poorer survival [34]. In addition, TFR is emerging as an important therapeutic goal in selected patients with CML. For those patients who have the ultimate goal of TFR, achieving sustained levels of deep molecular response is a prerequisite for attempting treatment discontinuation [7, 35].

Prior to ASC4FIRST, the ASCEMBL study had already demonstrated the favorable safety profile of asciminib compared to the 2G TKI bosutinib in heavily pretreated patients, which was shown in both the overall study population and Japanese patients from the study, and was maintained in the long-term [22, 23]. In general, the overall safety profile of asciminib in this Japanese subgroup of newly diagnosed patients was consistent with that observed in the overall population in ASC4FIRST [29]. For Japanese patients who received asciminib, there were no AEs leading to discontinuation, fewer grade ≥ 3 AEs, and fewer AEs leading to dose adjustment/interruption than observed with IS-TKI. Additionally, no new safety signals were found in this subgroup analysis. In summary, asciminib has a favorable safety and tolerability profile in Japanese patients, which is likely to enable patients to remain on treatment. This was evident in the current study, as by data cutoff, more patients had received at least 48 weeks of exposure to asciminib than IS-TKI (90.5% and 70.6%, respectively). Ultimately, longer exposure to treatment may lead to improved outcomes and a reduced need to switch treatment. Notably, the asciminib dose used in ASC4FIRST (80 mg QD) differs from the dose (40 mg BID) approved for the treatment of patients with resistant/intolerant CML in Japan [28]. However, in an analysis of the data from two asciminib studies conducted in patients with CML who had received at least two previous TKIs, the doses of 40 mg BID and 80 mg QD asciminib (80 mg total daily dose), had comparable efficacy, safety, and pharmacokinetics results [36]. In the phase III ASC4OPT study, these asciminib regimens (80 mg QD and 40 mg BID) were shown to be efficacious and well tolerated in previously treated patients [37]. Both dosage regimens are approved in the USA [27, 30]. A once daily dosing regimen may be more convenient to patients than the twice daily regimen used in the ASCEMBL study [21, 24], and could potentially improve adherence to treatment. The regulatory review is ongoing with an application for 80 mg QD asciminib for newly diagnosed CML based on the results of the ASC4FIRST study in Japan.

Hematological AEs are frequently observed with TKIs but there are differences in the profile of specific events observed with different TKIs [38]; as expected, hematological AEs were common in ASC4FIRST. Thrombocytopenia and neutropenia were the most frequent AEs observed in both the Japanese subgroup and overall population [29]; however, numerically higher incidences of these events occurred in Japanese patients, which may be a consequence of small patient numbers and inter-patient variability in this subset. Notably, these events did not result in the discontinuation of asciminib in the Japanese population. The incidences of grade ≥ 3 events for these hematological AEs were similar across asciminib and IS-TKI arms, and between the Japanese and overall populations [29]. For non-hematological AEs, the incidence of frequently observed AEs in Japanese patients was comparable to that in the overall population [29], with the exception of numerically higher rates of lipase increase for asciminib in the Japanese population compared to IS-TKI. However, none of these events resulted in treatment discontinuation, or dose adjustment or interruption in Japanese patients. Of note, there were no AOEs reported in the Japanese subgroup, while in the overall population very low incidences of AOEs in all groups (1.0%, 0%, and 2.0% for asciminib, imatinib, and 2G TKI, respectively) were observed [29].

Despite lower patient numbers in the Japan subgroup than in the overall population, the efficacy and safety outcomes observed in this Japanese subgroup were comparable with those in the larger, overall population, which showed the superior efficacy and favorable safety and tolerability profile of asciminib versus all current standard-of-care IS-TKI [29]. Collectively, these positive data from the Japanese subgroup analysis in patients with newly diagnosed CML-CP and the approval of asciminib for the treatment of patients with resistance or intolerance to previous TKI therapy, support asciminib as the potential therapy of choice for Japanese patients with CML-CP.

## Supplementary Information

Below is the link to the electronic supplementary material.

Supplementary file1 (DOCX 243 KB)

## Data Availability

Novartis is committed to sharing access to patient-level data and supporting clinical documents from eligible studies with qualified external researchers. These requests are reviewed and approved by an independent review panel based on scientific merit. All data provided are anonymized to respect the privacy of patients who have participated in the trial in line with applicable laws and regulations. This trial data availability is according to the criteria and process described on www.clinicalstudydatarequest.com.
