# Supplementary material for: Asciminib in patients with newly diagnosed chronic myeloid leukemia: results from the Japanese subgroup of ASC4FIRST
Source: Int J Hematol. 2025 May 26;122(5):647–59. doi: 10.1007/s12185-025-04014-z (PMC12572060; doi:10.1007/s12185-025-04014-z)
Supplement: Supplementary file 1 — Supplementary file1 (DOCX 243 KB) [file 12185_2025_4014_MOESM1_ESM.docx]

**Asciminib in patients with newly diagnosed chronic myeloid leukemia: results from the Japanese subgroup of ASC4FIRST**

Naoto Takahashi, Yoshikane Kikushige, Hirohisa Nakamae, Tatsunori Goto, Akihiro Tomita, Michiko Ichii, Satoshi Ito, Takanori Teshima, Keita Kirito, Takayuki Ikezoe, Kaoru Hatano, Hirokazu Tanaka, Nobuhiro Hiramoto, Ryohei Osako, Makoto Aoki, Kamel Malek, Yasunori Ueda

**Supplementary Fig. 1** Study design

**
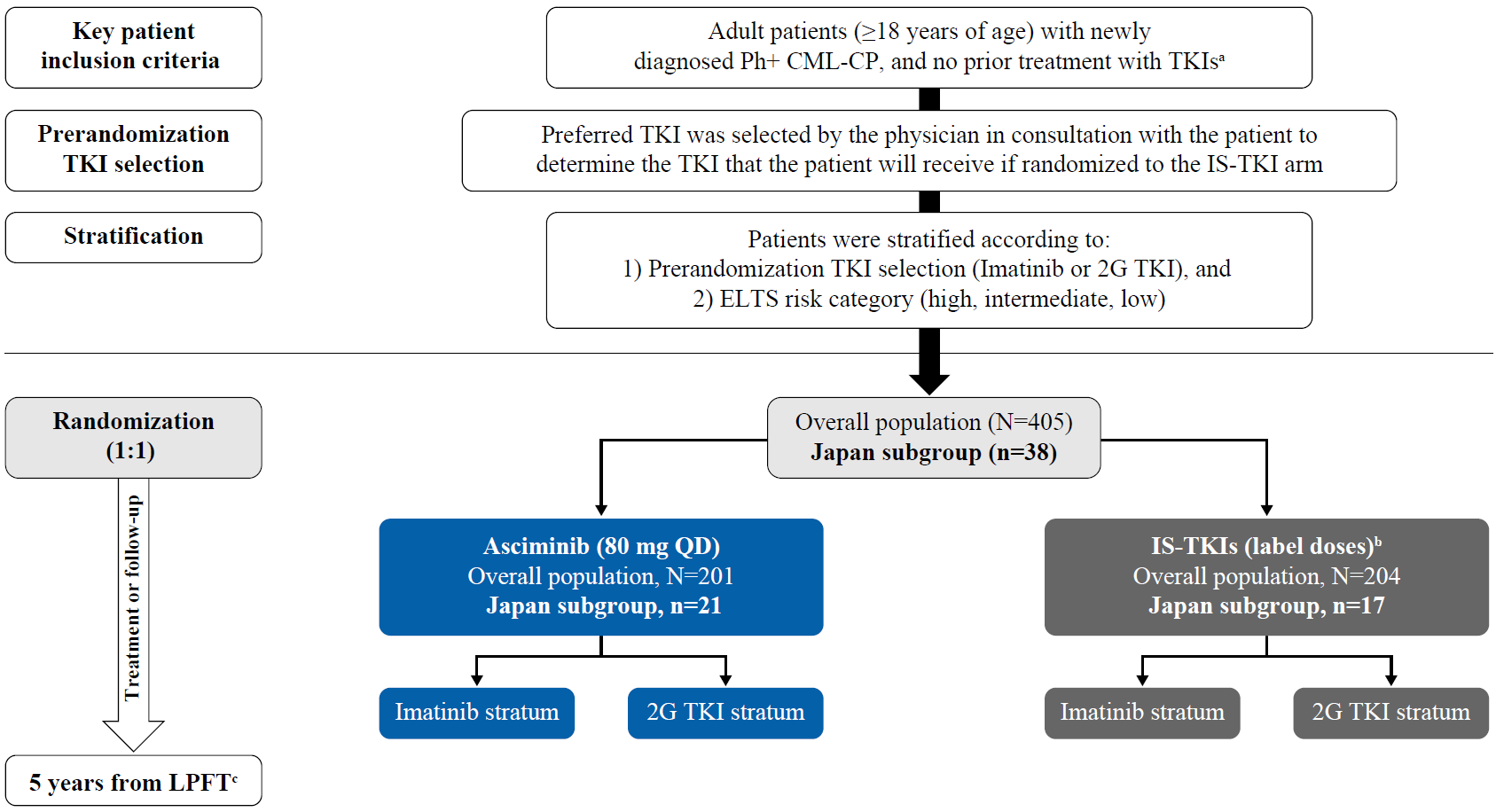
**

2G, second generation; BID, twice daily; CML-CP, chronic myeloid leukemia in chronic phase; ELTS, EUTOS long-term survival score; EUTOS, European Treatment and Outcome Study; IS-TKI, investigator-selected tyrosine kinase inhibitor; LPFT, last person first treatment; Ph, Philadelphia chromosome; QD, once daily; TKI, tyrosine kinase inhibitor.  ^a^Either imatinib, bosutinib, dasatinib, or nilotinib was allowed for up to 2 weeks prior to randomization, but treatment with other TKIs prior to randomization was not permitted;  ^b^Imatinib 400 mg QD, bosutinib 400 mg QD, dasatinib 100 mg QD, or nilotinib 300 mg BID; ^c^Patients will remain on study for 5 years after the LPFT, unless they have discontinued early (due to treatment failure, disease progression, pregnancy, intolerance, or investigator or patient decision). Patients who discontinued treatment will be followed until the end of the study.

**Supplementary Table 1.** Baseline demographics and characteristics

| **Baseline characteristics**  Categories | **Asciminib** | | | **IS-TKI** | | |
| --- | --- | --- | --- | --- | --- | --- |
|  | Imatinib stratum  (n=10) | 2G TKI stratum  (n=11) | All asciminib  (n=21) | Imatinib stratum  (n=8) | 2G TKI stratum  (n=9) | All comparators  (n=17) |
| **Age, years** | | | | | | |
| Mean (SD) | 61.4 (10.70) | 44.5 (16.26) | 52.5 (16.09) | 58.6 (14.07) | 55.2 (11.23) | 56.8 (12.36) |
| Median | 60.5 | 54.0 | 57.0 | 59.0 | 54.0 | 54.0 |
| Q1–Q3 | 54.0–72.0 | 28.0–58.0 | 41.0–61.0 | 46.0–71.0 | 48.0–65.0 | 47.0–68.0 |
| Minimum–maximum | 41.0–74.0 | 18.0–61.0 | 18.0–74.0 | 41.0–76.0 | 42.0–72.0 | 41.0–76.0 |
| **Age group, n (%)** | | | | | | |
| 18 to <65 years | 6 (60.0) | 11 (100) | 17 (81.0) | 4 (50.0) | 6 (66.7) | 10 (58.8) |
| 65 to <75 years | 4 (40.0) | 0 | 4 (19.0) | 3 (37.5) | 3 (33.3) | 6 (35.3) |
| ≥75 years | 0 | 0 | 0 | 1 (12.5) | 0 | 1 (5.9) |
| **Sex, n (%)** |  |  |  |  |  |  |
| Male  Female | 9 (90.0) 1 (10.0) | 7 (63.6)  4 (36.4) | 16 (76.2)  5 (23.8) | 5 (62.5)  3 (37.5) | 8 (88.9)  1 (11.1) | 13 (76.5)  4 (23.5) |
| **BMI, kg/m^2^** | | | | | | |
| Mean (SD) | 23.5 (2.68) | 22.5 (2.38) | 23.0 (2.51) | 23.9 (3.26) | 23.2 (4.69) | 23.5 (3.97) |
| Median | 22.5 | 22.8 | 22.6 | 24.8 | 22.6 | 22.7 |
| Q1–Q3 | 21.9–25.7 | 20.6–24.8 | 21.5–24.8 | 22.0–25.1 | 19.6–25.0 | 21.7–25.0 |
| Minimum–maximum | 20.1–28.8 | 18.3–26.2 | 18.3–28.8 | 18.1–29.3 | 18.3–33.2 | 18.1–33.2 |
| **Framingham estimated 10-year CVD risk categories, n (%)** | | | | | | |
| Low (<10%) | 2 (20.0) | 6 (54.5) | 8 (38.1) | 3 (37.5) | 4 (44.4) | 7 (41.2) |
| Intermediate (10%– 20%) | 1 (10.0) | 4 (36.4) | 5 (23.8) | 3 (37.5) | 2 (22.2) | 5 (29.4) |
| High (≥20%) | 7 (70.0) | 1 (9.1) | 8 (38.1) | 2 (25.0) | 3 (33.3) | 5 (29.4) |
| **Charlson Comorbidity Index, n (%)** | | | | | | |
| 2 | 1 (10.0) | 3 (27.3) | 4 (19.0) | 2 (25.0) | 4 (44.4) | 6 (35.3) |
| 3 | 3 (30.0) | 5 (45.5) | 8 (38.1) | 2 (25.0) | 1 (11.1) | 3 (17.6) |
| 4 | 1 (10.0) | 3 (27.3) | 4 (19.0) | 1 (12.5) | 2 (22.2) | 3 (17.6) |
| 5 | 3 (30.0) | 0 | 3 (14.3) | 0 | 1 (11.1) | 1 (5.9) |
| 6 | 1 (10.0) | 0 | 1 (4.8) | 1 (12.5) | 1 (11.1) | 2 (11.8) |
| 8 | 0 | 0 | 0 | 2 (25.0) | 0 | 2 (11.8) |
| 9 | 1 (10.0) | 0 | 1 (4.8) | 0 | 0 | 0 |
| **Estimated 10-year survival, %^a^** | | | | | | |
| Mean (SD) | 44.2 (34.64) | 74.4 (14.54) | 60.0 (29.72) | 48.9 (41.43) | 63.2 (33.10) | 56.4 (36.78) |
| Median | 37.4 | 77.5 | 77.5 | 65.4 | 77.5 | 77.5 |
| Q1–Q3 | 21.4–77.5 | 53.4–90.1 | 53.4–77.5 | 1.1–83.8 | 53.4–90.1 | 21.4–90.1 |
| Minimum–maximum | 0.0–90.1 | 53.4–90.1 | 0.0–90.1 | 0.0–90.1 | 2.2–90.1 | 0.0–90.1 |
| **ECOG PS, n (%)** | | | | | | |
| 0 | 10 (100) | 10 (90.9) | 20 (95.2) | 7 (87.5) | 9 (100) | 16 (94.1) |
| 1 | 0 | 1 (9.1) | 1 (4.8) | 1 (12.5) | 0 | 1 (5.9) |
| **ELTS score (IRT), n (%)** | | | | | | |
| Low | 8 (80.0) | 9 (81.8) | 17 (81.0) | 6 (75.0) | 6 (66.7) | 12 (70.6) |
| Intermediate | 2 (20.0) | 0 | 2 (9.5) | 2 (25.0) | 3 (33.3) | 5 (29.4) |
| High | 0 | 2 (18.2) | 2 (9.5) | 0 | 0 | 0 |
| **Reason for TKI selection, n (%)** | | | | | | |
| Age | 3 (30.0) | 3 (27.3) | 6 (28.6) | 3 (37.5) | 0 | 3 (17.6) |
| Co-morbidities | 1 (10.0) | 0 | 1 (4.8) | 0 | 0 | 0 |
| Local practice/ guidelines / clinical experience | 3 (30.0) | 6 (54.5) | 9 (42.9) | 1 (12.5) | 6 (66.7) | 7 (41.2) |
| Other | 1 (10.0) | 1 (9.1) | 2 (9.5) | 0 | 2 (22.2) | 2 (11.8) |
| Risk category | 2 (20.0) | 1 (9.1) | 3 (14.3) | 3 (37.5) | 0 | 3 (17.6) |
| Treatment objective | 0 | 0 | 0 | 1 (12.5) | 1 (11.1) | 2 (11.8) |

2G, second generation; BMI, body mass index; CVD, cardiovascular disease; ECOG PS, Eastern Cooperative Oncology Group performance status; ELTS, EUTOS long-term survival score; EUTOS, European Treatment and Outcome Study; IRT, interactive response technology; IS-TKI, investigator-selected tyrosine kinase inhibitor; n, number of patients; SD, standard deviation; Q, quartile
^a^Estimated 10-year survival based on Charlson comorbidity index.

**Supplementary Table 2.** Overview of AE profile across the arms and strata

2G, second generation; AE, adverse event; IS-TKI, investigator-selected tyrosine kinase inhibitor; n, number of patients; SAE, serious AE. Safety data presented for the ‘on treatment’ period (day of first administration to 30 days after the last administration of study treatment). A patient with mutiple severity grades for an AE is counted under the maximum grade. Medical Dictionary for Regulatory Activities (MedDRA; version 26.1); Common Terminology Criteria for Adverse Events (CTCAE; version 5.0).

| Category, n (%) | Asciminib | | IS-TKI | | | | | |
| --- | --- | --- | --- | --- | --- | --- | --- | --- |
|  | All asciminib  (n=21) | | Imatinib  (n=8) | | 2G TKI  (n=9) | | All comparators  (n=17) | |
|  | All grades | Grade ≥3 | All grades | Grade ≥3 | All grades | Grade ≥3 | All grades | Grade ≥3 |
| AEs | 21 (100) | 9 (42.9) | 8 (100) | 4 (50.0) | 9 (100) | 5 (55.6) | 17 (100) | 9 (52.9) |
| Treatment-related AEs | 16 (76.2) | 7 (33.3) | 8 (100) | 3 (37.5) | 9 (100) | 4 (44.4) | 17 (100) | 7 (41.2) |
| SAEs | 2 (9.5) | 1 (4.8) | 1 (12.5) | 0 | 1 (11.1) | 1 (11.1) | 2 (11.8) | 1 (5.9) |
| Treatment-related SAEs | 0 | 0 | 0 | 0 | 0 | 0 | 0 | 0 |
| Fatal SAEs | 0 | 0 | 0 | 0 | 0 | 0 | 0 | 0 |
| Treatment-related fatal SAEs | 0 | 0 | 0 | 0 | 0 | 0 | 0 | 0 |
| AEs leading to discontinuation | 0 | 0 | 3 (37.5) | 1 (12.5) | 1 (11.1) | 1 (11.1) | 4 (23.5) | 2 (11.8) |
| Treatment-related AEs leading to discontinuation | 0 | 0 | 2 (25.0) | 1 (12.5) | 1 (11.1) | 1 (11.1) | 3 (17.6) | 2 (11.8) |
| AEs leading to dose adjustment/ interruption | 7 (33.3) | 6 (28.6) | 5 (62.5) | 2 (25.0) | 6 (66.7) | 4 (44.4) | 11 (64.7) | 6 (35.3) |
| AEs requiring additional therapy | 17 (81.0) | 3 (14.3) | 8 (100) | 1 (12.5) | 9 (100) | 4 (44.4) | 17 (100) | 5 (29.4) |
